# Multi-Parameter Optimization of 3D Printing Condition for Enhanced Quality and Strength

**DOI:** 10.3390/polym14081586

**Published:** 2022-04-13

**Authors:** Brandon Jackson, Kamran Fouladi, Babak Eslami

**Affiliations:** Mechanical Engineering Department, Widener University, Chester, PA 19013, USA; bojackson@widener.edu (B.J.); kfouladi@widener.edu (K.F.)

**Keywords:** additive manufacturing, optimization, 3D printing, fused depositional modeling

## Abstract

Fused deposition modeling (FDM) 3D printing is the most common type of additive manufacturing available in both research and the industry. Due to the rapid development of 3D printing, there is now a significant need to fabricate parts with *higher quality* with respect to cosmetics, precision, and strength of the final products. This work is focused on finding the optimal printing condition for a commercially available 3D printer and filament material (i.e., Polylactic acid (PLA)). In this work, we focus on finding the combined effect of retraction speed, deposition angle, and number of walls on both the visual quality and strength of 3D-printed parts. It is found that the number of walls does not play a major role in the strength of the parts. On the other hand, the retraction speed plays a significant role in defining the ultimate tensile strength of the parts. For parts printed at higher retraction speeds, there is a 10–15% improvement in the ultimate tensile strength.

## 1. Introduction

Advancements in Fused Deposition Molding (FDM), widely known as 3D printing, have become more abundantly widespread for industrial use and commercial sale. Three-dimensional printing works with polymers such as Polylactic acid (PLA), Acrylonitrile butadiene styrene (ABS), and Polyethylene terephthalate glycol (PTEG). It is accomplished by heating the polymer and by depositing it one layer at a time to build a 3D model based on the given design. These models are typically designed in Computer-Aided Design (CAD) software and then are sliced into a G-code, where printing parameters are defined and then uploaded to a printer to fabricate the part/s [1]. The primary industrial usage for 3D printing is prototyping. However, different fields such as aerospace, automotive, healthcare, and general product development-related applications have found ways to use 3D printing to fabricate final products for their needs [2].

With this rapid development of 3D printing, the printing process to generate higher “quality” products must become profoundly more efficient with optimal print settings and without repeated unwanted mistakes. However, quality in 3D printing is not well defined, and there is no universal standard for quality. Past research has mainly focused on the dimensional accuracy, level of detail on the surface, and bridging as measures of quality [3,4]. Moreover, it has been argued that aspects such as surface quality can directly affect the dimensional accuracy and functionality of the part while negatively impacting the visual aesthetic [5]. However, finding the correct parameters for surface quality can be challenging. Past studies have investigated the impacts of various parameters on print quality. These parameters include printing support, raster angle, extruder temperature, layer thickness, print orientation, layer height, infill percentage, fill pattern, and the number of walls/shells.

Printing with supports added to a structure during the fabrication process can lead to material waste, longer print time, and surface damage during removal. Moreover, printing with supports usually requires more advanced 3D printers with multiple hot-ends. Printing without support is an alternative to overcoming these challenges to reduce these issues. Without using supports, researchers have been able to reduce the print time by up to 20.6% and the material usage by 22.33% for structures with clear block geometries due to their decomposition process [4]. However, unsupported printing poses its own challenges if the retraction speed, temperature of the extruder, distance over open space, and movement speed are not properly set. This is due to the fact that unsupported prints for overhung parts rely on the geometry and bonding strength of each element during the print. For example, if a dome is printed with an unsupported structure, the retraction speed and deposition angle play major roles in the success of the part. Otherwise, the filament is deposited too quickly at high angles and the material does not bond. The improper setting of these parameters can affect the quality by changing how the parts look compared with the original design in the form of stringing or oozing, which is small strings of plastic left behind between unsupported gaps [6]. A proper extruder temperature is also crucial for achieving parts dimensional accuracy. For example, it is found that temperatures of 220 °C as a maximum temperature for PLA and minimum temperature for ABS provide better dimensional accuracy [7]. This is mainly due to the fact that higher print temperatures dispense the polymer at higher concentrations of the liquid–solid mixture and consequently require longer periods of time to solidify. Since this time is not provided due to the retraction speed of the print, the parts are not printed accurately.

Parameters such as print orientation, raster angle, layer height, infill percentage, fill pattern, and the number of walls/shells have also been observed to significantly impact the ultimate tensile strength. Print orientation, the alignment of the printed part within the build space, has been shown to drastically change ultimate tensile [8]. For 100% infill percentage, Nylon 960 has the highest ultimate tensile strength of 69 MPa while PLA has the weakest at 33 MPa [9]. Moreover, substantial improvements in ultimate strength were achieved for the ABS material when printed at 100% infill compared with lower infill percentages in the rectangular patterns [10]. Parts printed at 100% infill generally achieve higher ultimate tensile strength since a higher surface area for bonding to occur between each layer is achieved.

The raster angle plays a vital part in quality measures such as dimensional accuracy and ultimate strength. The effects of raster angle on dimensional accuracy for printing with PLA have been presented by Tontowi et al. [11]. They also investigated the role of raster angle on ultimate strength. They showed that parts printed at a raster angle of 45° result in higher tensile strength than 0 or 90°. Moreover, changing the angle at which the part is printed can also directly affect the surface quality, especially in situations where the overhang is present [12].

Similar to raster angle, the layer thickness can also affect the quality when printing with PLA [3,13]. More specifically, layer thickness significantly impacts the tensile strength of PLA 3D-printed parts [11]. The number of walls/shells is another parameter that has been investigated for print quality. Studies such as [8,14] have investigated the effects of the number of walls/shells on tensile strength in comparison with the effects of infill percentage. They have argued that the number of walls/shells may impact tensile strength more significantly than the infill percentage because they carry more of the load. While this has not been proven conclusive, there is good reasoning that the bond of the shells increases the structural integrity of the print without leaving spaces that cause instability where it is bonded with the infill. It should also be noted that, although there are different polymers available to do FDM 3D printing, PLA is the most common type. PLA is used in different fields in addition to 3D printing such as injection molding, thermoforming, and fiber spinning [15,16]. PLA polymer is biodegradable and recyclable since its backbone ester group goes through hydrolysis and progresses during its degrading process. Additionally, PLA is biocompatible with the body fluids and, therefore, can be used in biomedical applications. It does not produce toxic effects in local tissues, which adds to the 3D printing value for bio-related devices [17,18,19,20,21].

It should be noted during the past several years, there has been an extensive amount of effort put into the field of FDM 3D printing to first understand the influence of printing parameters on parts and second to develop a methodology to perform quality control on parts. For example, Cwikla et al. have studied the influence of infill pattern and infill density on 3D-printed parts [22]. Buj-Corral et al. have studied the influence of print orientation on the surface roughness of FDP parts [23]. Vanaei et al. have developed a numerical model to predict the profile of 3D-printed parts [24]. By using their model, one can improve the bonding strength of parts by having a better understanding of the temperature evolution of deposited filaments. There are also advanced characterization techniques used to study the quality of 3D-printed parts [25]. However, none of the above studies have performed a comprehensive investigation on the trade-offs of each of the printing parameters and their influence on both the quality of the 3D-printed parts and their strengths.

### Objective and Motivation

A review of published work points to the lack of universal standards for print quality. Among the many parameters proposed and studied to establish print quality standards are dimensional accuracy, level of surface detail, bridging, and tensile strength. Moreover, many different printing setup parameters have been investigated in these studies for their impact on print quality. However, any parameter considered in these studies has been studied individually, although it is evident that each printing parameter can have a trade-off effect on the other. Therefore, a generalized strategy is needed for evaluating various print quality metrics, while impacts of print setup parameters are investigated.

The present study is focused on developing a quality control procedure and performing a multi-parameter study to find the optimal FDM 3D printing condition for PLA to provide the best (1) surface finish, (2) bridging in unsupported areas, (3) dimensional accuracy, and (4) strength of the 3D-printed part. There are three major printing parameters that will be modulated in this study *simultaneously*: number of walls (i.e., shells), retraction speed, and printing orientation from the *z*-axis.

## 2. Materials and Methods

**Figure 1:**
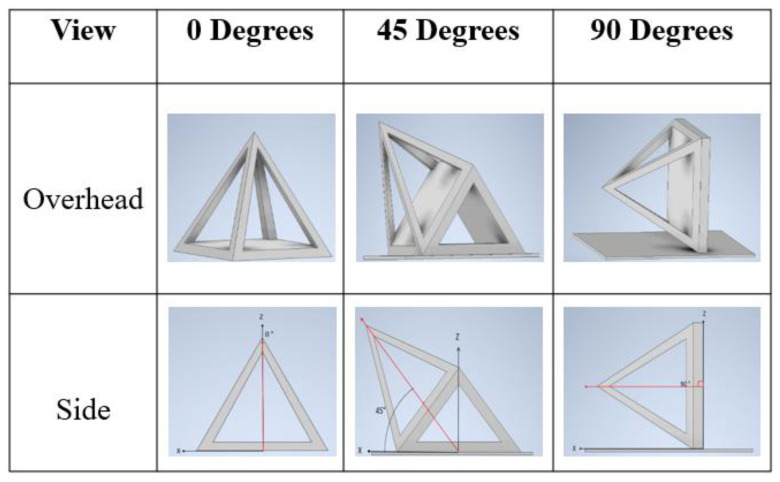
CAD models of open pyramid samples with different orientations of 0°, 45°, and 90°.

*Sample Design*: AutoDesk Inventor^®^ was used to create a 3D CAD model to test the qualitative aspects of the print: surface quality and unsupported printing quality. Figure 1 provides different orientations of various pyramid samples. The overall dimensions of the pyramids were 40 mm for each of the side lengths and height. The pyramid sample set was designed and printed for the quality control tests. They were also selected due to their printing challenges as they require all three axes to work simultaneously in addition to having unsupported material.

**Figure 2:**
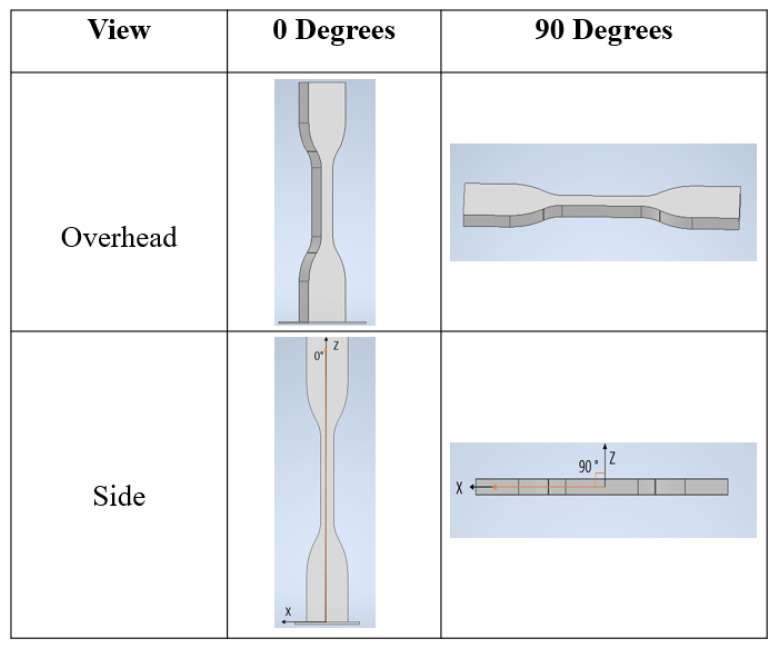
CAD model of dog bone samples with 0° and 90°.

The dog bone samples were designed in Autodesk Inventor following ASTM D638 standards to test the tensile characteristics on PLA with changing parameters listed in Figure 2. The printer used for this experiment was Mantis 3D Printer manufactured by Verde Mantis, LP (Reading, PA, USA). Mantis is generally considered a user-friendly printer due to its internal programming as it automatically selects the most optimal printing orientation. However, the present study aimed to investigate different orientations. Therefore, different bases were constructed to work around this automated programming and to obtain that desired orientation from the *z*-axis.

*Sample Preparation*: There were nine individual g-codes for each sample of the varying angles of the open pyramids that were uploaded to Mantis prior to each print. This resulted in 27 prints split between the 0°, 45° and 90° variations. The same process was repeated for the dog bones with the nine individual G-code printing parameters. This resulted in 18 different samples ranging between the 0° and 90° degrees. The G-Code parameters for each of the samples are listed in Table 1.

For the pyramids, the samples were printed in three different orientations with three different sets of retraction speeds and three wall thicknesses. The dog bone samples were printed in two different orientations with three different retraction speeds and three wall thicknesses. The parameters that were altered for the G-Code for the open pyramid samples and dog bone samples are shown in Table 2 and Table 3.

*Surface Quality*: The surface roughness on prints depends on the change in height between the constructed layers and the gaps between them. A direct parameter that could cause a change in surface quality would be the printing orientation of the surface. The filament placement can become obstructed due to possible limitations in the programming for specific geometrical structures that could cause unwarranted gaps, ripples, and bumps. This effect has been proven in areas where a drastic overhang is present. Therefore, determining the angle causing the greatest digression in surface quality can be helpful at the design stage to prevent this quality issue during printing. The area of the top base on the open pyramid samples is used to examine the surface quality of each print. The sense of touch is an excellent indicator to compare roughness since drastic changes in the layer height can be felt. The surface quality index in Table 4 was created to encapsulate the comparison of a smooth surface with a rougher surface for the samples presented in the study.

The ratings in Table 4 are based on subjective qualities since there is no universal standard of surface quality. However, lower surface quality has been shown to cause lower dimensional accuracy in prints [13,26]. Some of the aspects focused on surface quality are how rough or smooth the surface is, or whether there are any bumps or warped parts on the surface. The examples in Table 4 are selected from the samples that meet the criteria of their given rating as reference.

*Unsupported Printing Quality*: Oozing and stringing are the most apparent issues with unsupported printing. The amount of oozing and stringing in a 3D print indicates how much material is wasted and if the fabricated print looks similar to its intended CAD design specifications. A direct parameter that may affect this issue is the rate of retraction speed. Retraction speed is the rate at which the extruder pulls back on a filament. A low retraction speed can cause excess filament to be extruded during the fabrication process, resulting in slight to drastic differences between the printed part and its distinctive design. The long gaps between the legs of the open pyramids’ samples are designed to test the quality of unsupported printing, and the amount of stringing that occurs during the process. The evaluation criteria used as the rating index for the unsupported printing are presented in Table 5.

The ratings in Table 5 are based on subjective qualities that are inspired by previous research that found these properties to frequently give the best results when used for assembly. Some of the key aspects that are focused on are the amount and the length of stringing present on the structure. The examples in Table 5 are selected from the samples that meet the criteria of their given rating as reference.

**Figure 3:**
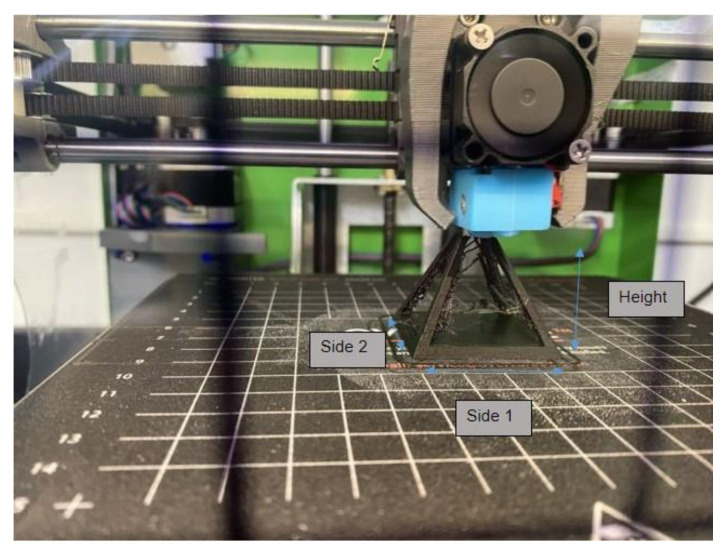
Dimensional layout description for 0° from the *z*-axis open pyramid sample.

*Dimensional Accuracy:* Two direct parameters that could alter the dimensional accuracy of the sample are the retraction speed and the printer position from the *z*-axis. The excess filament deposited due to lower retraction speed could increase the expected lengths of the printed samples. The overhang from the change in position of *z*-axis may cause the filament to fall during the print and causes a failed print. In the present study, digital calipers were used to measure both sides of the base and the height for the open pyramid sample for dimensional accuracy. Side 1 for each print was considered the side facing the opening of the 3D printer enclosure. Therefore, the orientation of samples was never changed since all CAD files were the same. Side 2 was then considered the opposing length of Side 1. The height was always measured from underneath the base of the open pyramid to the pointed tip of each sample. A visual description for the sides is displayed in Figure 3.

**Figure 4:**
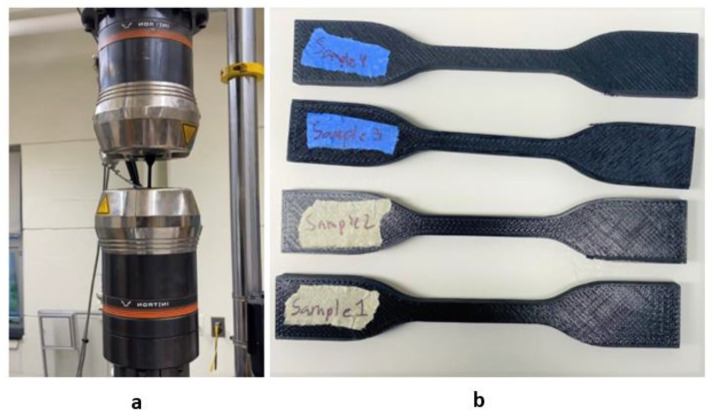
Tensile Testing for dogbone samples: (**a**) Instron Tensile Tester, (**b**) set of 3D printed dogbone samples according to ASTM standard.

*Tensile Testing:* ASTM dog bone samples were also created to test the ultimate tensile strength with changing parameters. Two direct parameters that could alter tensile testing are the number of walls and the retraction speed. It has been shown in previous research that ultimate tensile strength increases as the number of walls increases. The slow retraction speed can cause an excess filament to be extruded, causing certain sections to be slightly disproportional. That disproportion could affect the bonding between the infill and the walls if the raster angle changes, resulting in a change of strength. An example of the infill pattern is presented in Figure 4b. All tensile tests were performed on the Instron Tensile Tester with the strain rate of 0.02 in/s, where each dog bone was pulled until fracture to gather a complete range of tensile strength vs. elongation of each sample displayed in Figure 4a.

## 3. Results

**Figure 5:**
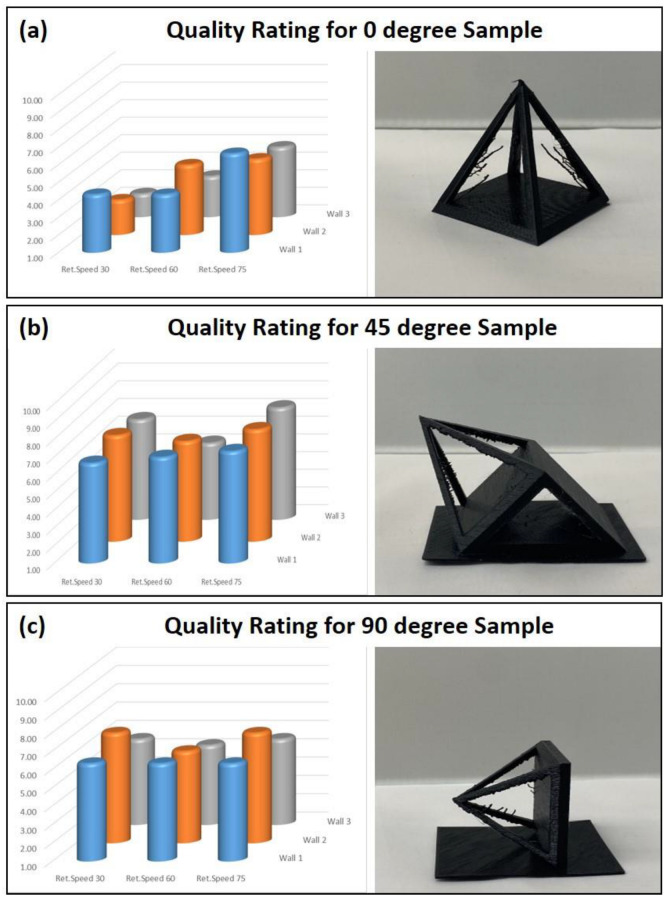
Quality ratings: (**a**) 0° printing orientation, (**b**) 45° printing orientation, and (**c**) 90° printing orientation. The x-axis shows the three retraction speeds: 30, 60, and 75 mm/s. The y-axis shows the three wall thicknesses: 1, 2, and 3.

*Quality Control:* Two metrics were discussed in the methods: surface quality (out of 5) and unsupported print quality (out of 5) were combined into a single metric out of 10 to rank them based on their printing conditions. As discussed earlier, each orientation was printed with three different retraction speeds and three wall thicknesses, resulting in 27 individual prints. Figure 5 represents the summary of each score received for these samples.

The bar graphs in Figure 5 represent the quality score of the printed parts out of 10. If printed at 0°, the best quality was observed when printed at 75 mm/s retraction speed with a wall number of one. For a 45° print, the best quality is also seen at 75 mm/s regardless of the wall thickness. The best quality for 90° was achieved when printed at the slowest and fastest retraction speeds for a wall thickness of 2. As shown in Figure 5, a lower quality is observed in 0° prints. The retraction speed at 30 mm/s showed inadequate quality in the prints. As the retraction speed increased to 60 mm/s, the quality increased for all different deposition angles. The highest quality ratings are observed for the 45° deposition angle. Overall, the number of walls did not play a major role in the quality of the prints. The retraction speeds at both 30 mm/s and 75 mm/s were equal in overall quality at their respective wall counts except for a significant increase when two walls were present, while prints at 60 mm/s stayed relatively similar, showing no variation as the number of walls varied.

**Figure 6:**
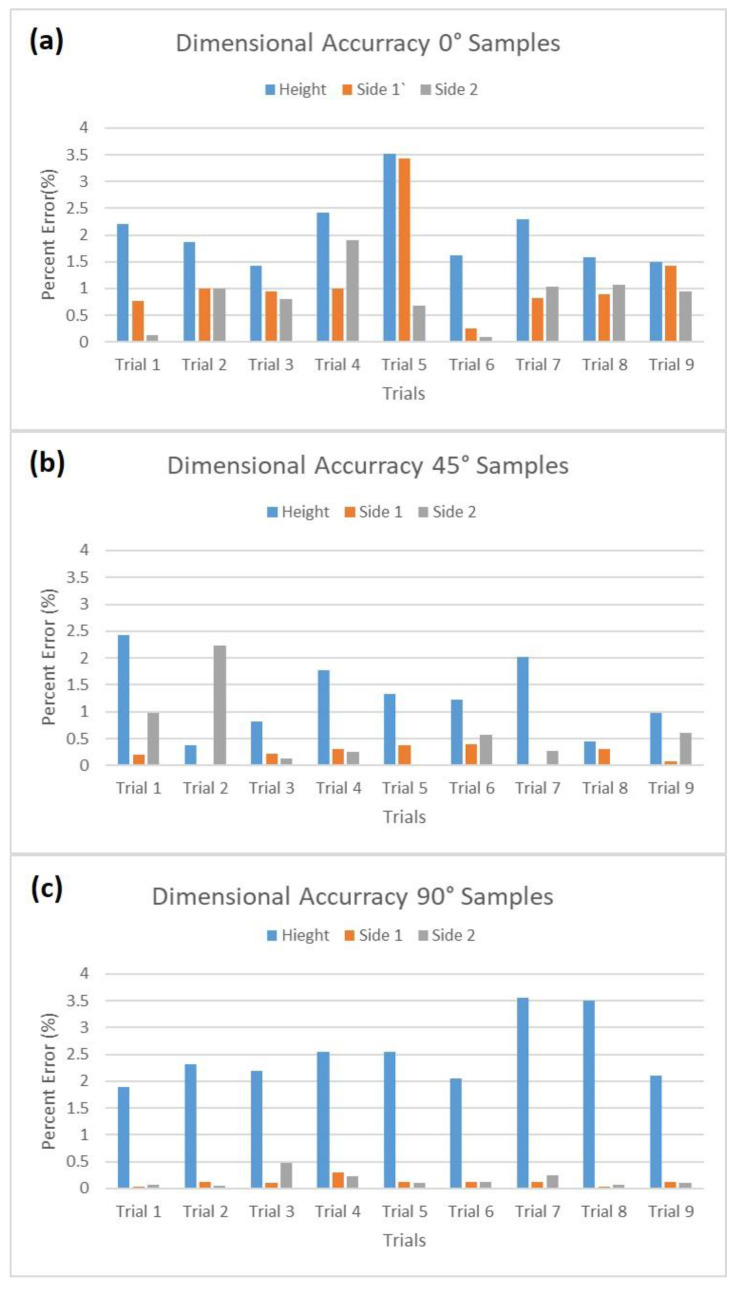
Percent error for three different sides of pyramid: (**a**) 0° deposit angle, (**b**) 45° deposit angle, and (**c**) 90° deposit angle. Blue, orange, and gray colors are for the height, side 1, and side 2, respectively.

*Dimensional Accuracy:* The first quantitative measurement for the 3D-printed parts was the dimensional accuracy of different print sides. The height and the two sides of the pyramid base are shown in Figure 3. Each dimension was compared with the theoretical value defined by the CAD model to obtain the percentage error. Figure 6 represents a set of data showing which printing configuration caused different levels of errors. The blue bar represents the percent error for height, and orange and gray bars represent the side 1 and 2 percent errors, respectively. Figure 6a–c present 0°, 45°, and 90°, respectively. As shown, in all prints, the highest error was shown in height. Moreover, the lowest error in all dimensions was achieved when the part was printed in 90°. This important finding can help users select the proper orientation during the slicing process when size is a curtail factor for their parts.

**Figure 7:**
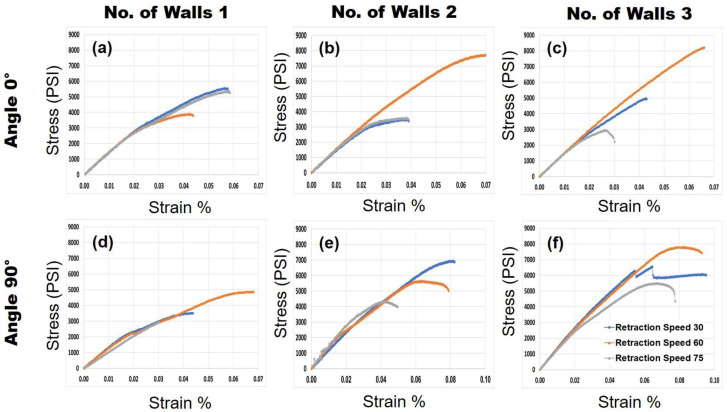
Stress–strain curves: (**a**–**c**) are for a deposition angle of 0° for 1, 2, and 3 walls, respectively. (**d**–**f**) are for a deposition angle of 90° for 1, 2, and 3 walls, respectively. The colors blue, orange, and gray refer to retraction speeds of 30, 60, and 75 mm/s, respectively.

*Tensile Testing:* The stress versus strain curves for each sample were gathered for different printing conditions. The three different retraction speeds for three different wall thicknesses were tested for both 0° and 90° samples. All of the stress–strain curves are shown in Figure 7. Based on the results shown in Figure 7, it can be seen that the elastic modulus and elongation of the samples are relatively similar to each other regardless of the printing conditions. However, the parameter that has changed is the ultimate tensile strength. In Figure 7, each column represents the number of walls while the rows represent the deposition angle. Each stress–strain curve includes three different retraction speeds of 30, 60, and 75 mm/s shown in blue, orange, and gray, respectively.

**Figure 8:**
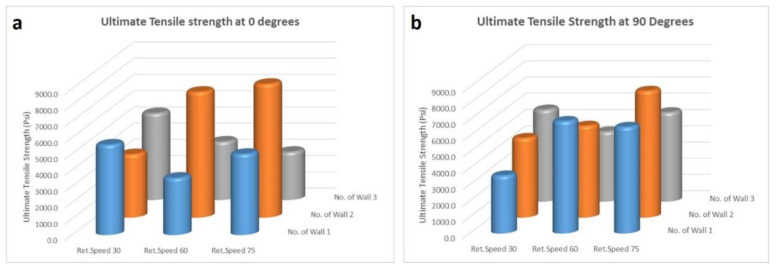
(**a**) Ultimate tensile strength at 0° and (**b**) ultimate tensile strength at 90°. The ultimate strength axis is from 0 to 9000 psi (equivalent to 0 to 62 MPa).

Figure 8 is presented to systematically study the effect of printing conditions on ultimate tensile strength. It is found based on Figure 8a that the highest ultimate tensile strength was observed when the number of walls was 2 with the traction speed of 75 mm/s. It was also shown that, when the wall thickness is two, the increase in retraction speed for either of the deposition angles increases the strength of the printed parts. Based on these trends, it is found that, as the retraction speed increases, there is generally an improvement in the ultimate tensile strength. This improvement can be due to the new layer of PLA polymer deposited on a previous one before they fully solidify, creating a stronger bond between layers. There is no conclusive trend between the ultimate tensile strength and the number of walls.

## 4. Conclusions

In this paper, multiple parameters that can be modified in processing the CAD file before starting the 3D print job were studied simultaneously. This study was focused on assessing qualitative and quantitative studies. The three main parameters under study are (1) retraction speed, (2) wall thickness, and (3) deposition angle. The quality of 3D-printed parts was measured by (1) surface quality, (2) dimensional accuracy, and (3) material strength. The dimensional accuracy testing at different positions found that samples printing at 45° from the *z*-axis had the least percent error for their measured lengths. The surface quality was heavily dependent on the retraction speed and angle from *z*-axis regardless of the number of walls. At high retraction speeds (i.e., 75 mm/s), the surface quality was observed to either slightly or significantly increase depending on the position from the *z*-axis. The orientation from the *z*-axis at both 45 and 90° displayed a high degree of consistency and quality compared with printing at 0°. Overall, 45° was the most optimal orientation for the high surface quality. For prints printed in the 0° and 45° orientations from the *z*-axis, there was a noticeable improvement in unsupported printing quality as the retraction speed increased. The overall quality was also heavily dependent on the retraction speed and position regardless of the number of walls present. In the orientation of 0 and 45°, the unsupported print quality increased as the retraction speed increased. The 90° orientation displayed minimal variation when the retraction speed or number of walls was altered. The tensile testing results found that the most optimal printing condition was when there were two walls at the 75 mm/s retraction speed. This condition achieved the highest ultimate tensile strength at both 0° from the *z*-axis with 8214.7 psi and 90° from the *z*-axis with 7797.3 psi.

## Figures and Tables

**Table 1:** Constant print setting parameters for the experiments.

Print Setting	Constant Print Settings
Bed Temperature	60 °C
Layer Height	0.1 mm
Extruder Temperature	215 °C
Shape	Rectangular
Infill	100%
Infill Speed	70 mm/s
Fill Angle	45°
Filament Type	PLA

**Table 2:** Printing configuration for open pyramids.

Angle 0°			Angle 45°			Angle 90°		
Sample	Retraction Speed (mm/s)	Walls	Sample	Retraction Speed (mm/s)	Walls	Sample	Retraction Speed (mm/s)	Walls
Sample 1	30	1	Sample 10	30	1	Sample 19	30	1
Sample 2	30	2	Sample 11	30	2	Sample 20	30	2
Sample 3	30	3	Sample 12	30	3	Sample 21	30	3
Sample 4	60	1	Sample 13	60	1	Sample 22	60	1
Sample 5	60	2	Sample 14	60	2	Sample 23	60	2
Sample 6	60	3	Sample 15	60	3	Sample 24	60	3
Sample 7	75	1	Sample 16	75	1	Sample 25	75	1
Sample 8	75	2	Sample 17	75	2	Sample 26	75	2
Sample 9	75	3	Sample 18	75	3	Sample 27	75	3

**Table 3:** Experimental trials for the dog bone samples.

Angle 90°			Angle 0°		
Samples #	Retraction Speed (mm/s)	Walls	Samples #	Retraction Speed (mm/s)	Walls
1	30	1	10	30	1
2	30	2	11	30	2
3	30	3	12	30	3
4	60	1	13	60	1
5	60	2	14	60	2
6	60	3	15	60	3
7	75	1	16	75	1
8	75	2	17	75	2
9	75	3	18	75	3

**Table 4:** Surface quality rating index.

Surface Quality Rating Index		Examples
**5**	No imperfection and no visible positive or negative (dips, ripples, or bums) spacing and markings on part that are not intentional to parts design to touch surface will feel like a smooth finish.	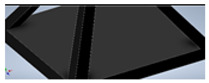
**4**	Very little imperfection like small bumps. Very little visible positive or negative spacing (dips, ripple, or bumps) spacing or marks that are not intentional to parts design. Surface will feel very close to a smooth finish.	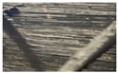
**3**	Small to moderate bumps. Slight markings that look off but don’t obscure the form of the overall structure greatly. Moderate visible positive or negative spacing (dips, ripples, or bumps). Surface will closely resemble intentional design but not perfectly. To touch surface may feel slight gritty but closely to smooth.	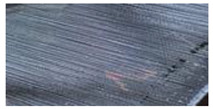
**2**	Slightly moderate bumps. Slight markings that look off and positive or negative spacing (dips, ripples, or bumps). Surface will have distorted slightly warped look. Surface may be slightly gritty like a finer sand paper. Visible markings that aren’t intentionally placed.	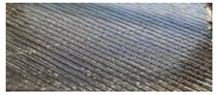
**1**	Very visible positive or negative spacing (dips, rippled, or bumps). Surface may feel warped not resembling intentional design. Larger marking that aren’t intentional. Surface may feel very gritty almost like rough sand paper.	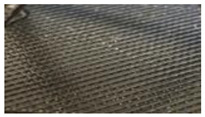

**Table 5:** Unsupported printing quality rating index.

Unsupported Printing Quality Rating Index		Examples
**5**	No stringing visible around structure.	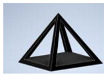
**4**	Light stringing covering structure. Small in places does not stretch far. Little build up. Up to 3 small string branches.	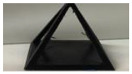
**3**	Medium stringing abundantly showing. Structure looks noticeably different from design. Stretches out. Some build up in certain spots. 4–7 small string branches.	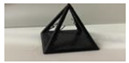
**2**	Abundant stringing reaching across places like webbing. Build up warps structure shape. 7–12 long stretched out stringing branches.	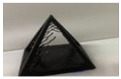
**1**	Structure is covered mainly in web like stringing all over. Build up makes structure not look like intended design. 12+ long stringing branches.	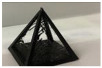

## Data Availability

Not applicable.
